# Prenatal Detection of Intra‐Abdominal Cyst Leading to Neonatal Diagnosis of OHVIRA Syndrome

**DOI:** 10.1111/ped.70551

**Published:** 2026-09-24

**Authors:** Kurumi Narusawa, Yoshio Shima, Tsubasa Takahashi

**Affiliations:** ^1^ Department of Neonatal Medicine Nippon Medical School, Musashikosugi Hospital Kawasaki Japan; ^2^ Department of Pediatric Surgery Nippon Medical School, Musashikosugi Hospital Kawasaki Japan

**Keywords:** early diagnosis, multidisciplinary follow‐up, OHVIRA syndrome

Obstructed hemivagina and ipsilateral renal anomaly (OHVIRA) syndrome is a rare congenital disorder characterized by uterine duplication, unilateral obstructed hemivagina, and ipsilateral renal anomalies. It is characterized by the co‐occurrence of urinary and genital tract anomalies on the same side [1]. OHVIRA syndrome is typically diagnosed after puberty because affected patients commonly present with dysmenorrhea or pelvic pain. However, recent advances in prenatal and postnatal imaging techniques have increased opportunities for earlier diagnosis. We report a neonatal case of OHVIRA syndrome that was incidentally diagnosed during the evaluation of an intra‐abdominal cyst detected on prenatal ultrasonography.

A female infant was born at 41 weeks and 2 days of gestation by vaginal delivery to a 39‐year‐old mother following an uncomplicated pregnancy achieved through in vitro fertilization. Her birth weight was 2946 g. Fetal ultrasonography performed at 22 weeks of gestation demonstrated an intra‐abdominal cyst, which showed no change in size or appearance during pregnancy (Figure 1A). Visualization of the right kidney was consistently limited on fetal ultrasonography because of the fetal position. Therefore, a prenatal diagnosis of right renal agenesis was deferred. No apparent abnormalities were identified on postnatal physical examination. Postnatal abdominal ultrasonography revealed a 2‐cm unilocular cyst adjacent to the bladder. Additionally, right renal agenesis was identified, whereas the left kidney appeared normal. Magnetic resonance imaging performed at 11 days of age demonstrated a fluid‐filled cyst, likely due to unilateral obstruction of the hemivagina associated with uterine didelphys (Figure 1B,C). Concurrently, ipsilateral renal agenesis was confirmed (Figure 1D). Based on these findings, the infant was diagnosed with OHVIRA syndrome. As the patient was asymptomatic, surgery was deferred, and a multidisciplinary follow‐up plan involving pediatricians, urologists, and gynecologists was established, with periodic imaging for long‐term monitoring.

**FIGURE 1:**
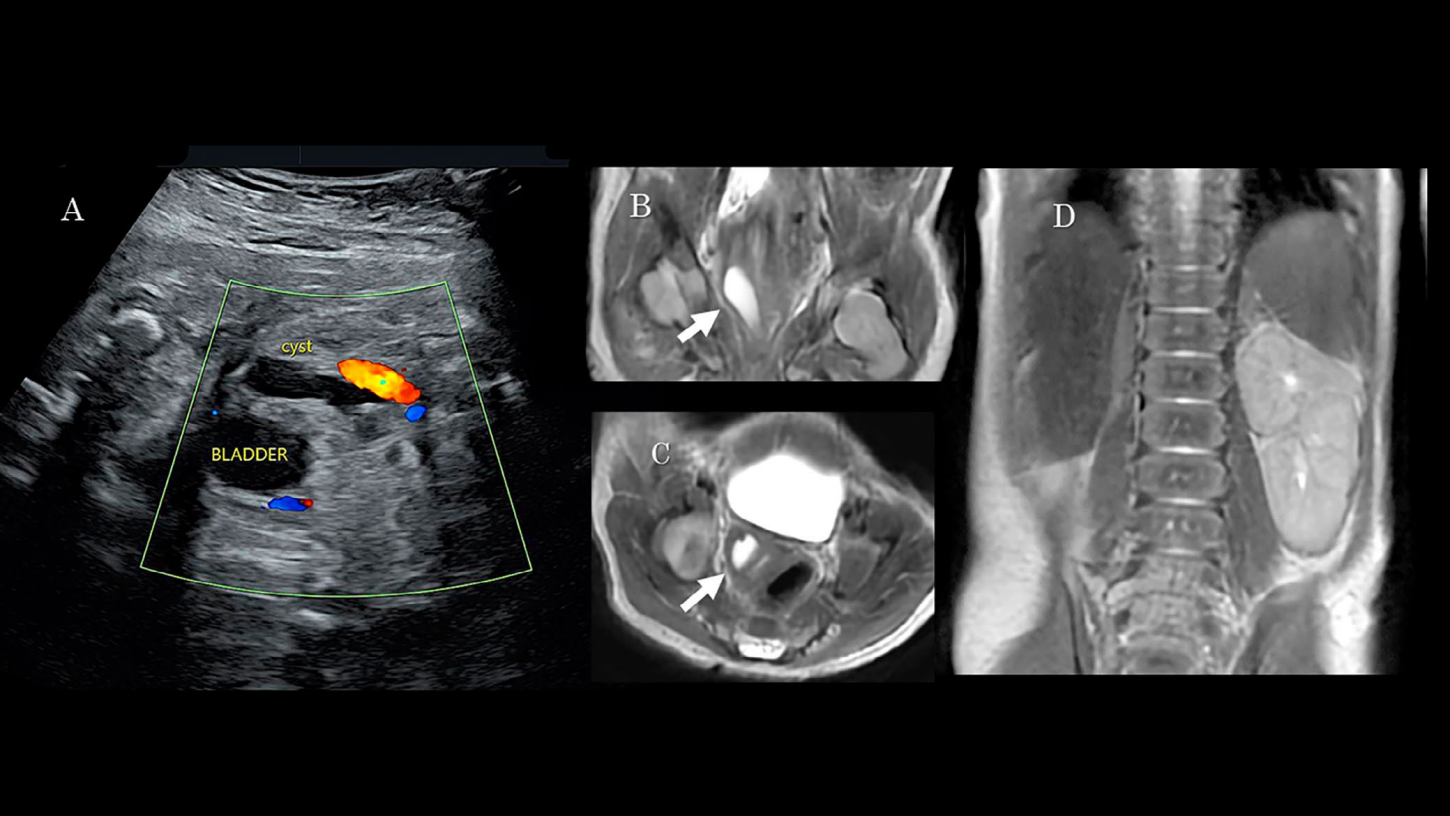
Fetal ultrasonography showing a unilocular cyst adjacent to the bladder (A). Pelvic magnetic resonance imaging obtained at 11 days of age. T2‐weighted coronal (B) and axial (C) images demonstrating a markedly dilated fluid‐filled cavity, indicative of obstructed hemivagina associated with uterus didelphys. Ipsilateral renal agenesis is evident (D).

OHVIRA syndrome resulted from abnormal development of the Müllerian and Wolffian ducts, causing ipsilateral reproductive and urinary tract anomalies. Because the mesonephric duct is essential for both systems, renal agenesis or dysplasia may indicate the presence of associated genital tract malformations [1]. However, diagnosis is often delayed until adolescence, as symptoms such as dysmenorrhea, pelvic pain, or recurrent genital tract infections typically appear only after menarche as a consequence of outflow obstruction [2]. The present case highlights two key clinical points.

First, prenatal or early postnatal detection of a pelvic cystic lesion in a female infant with ipsilateral renal agenesis should prompt careful assessment for associated Müllerian anomalies, such as OHVIRA syndrome. Although neonatal diagnosis remains uncommon, advances in fetal ultrasonography and postnatal magnetic resonance imaging have increased opportunities for early detection before symptom onset [3]. In the present case, persistent prenatal identification of an intra‐abdominal cyst prompted targeted postnatal imaging, enabling diagnosis during the neonatal period despite the absence of clinical manifestations.

Second, early diagnosis may have important implications for long‐term management. Delayed recognition of OHVIRA syndrome may lead to complications such as hematocolpos, endometriosis, pelvic adhesions, infection, and impaired fertility [2]. Although the optimal timing of surgery in asymptomatic patients remains controversial, establishing the diagnosis before puberty allows longitudinal follow‐up and anticipatory counseling for patients and their families regarding future gynecologic and reproductive outcomes [4]. In this context, neonatal diagnosis may facilitate timely intervention if obstructive symptoms develop.

In conclusion, OHVIRA syndrome should be considered in the differential diagnosis of female neonates with renal agenesis and ipsilateral pelvic cystic lesions. Early recognition of this association may enable timely diagnosis and appropriate multidisciplinary follow‐up before complications arise.

## Author Contributions

K.N. drafted the manuscript draft, Y.S. revised the manuscript, and T.T. provided patient care. All authors have read and approved the final version of the manuscript.

## Funding

The authors have nothing to report.

## Disclosure

The authors have nothing to report.

## Consent

Informed consent for publication of this report was obtained from the patient's parents. The patient's records and personal information were anonymized to protect privacy.

## Conflicts of Interest

The authors declare no conflicts of interest.

## Data Availability

The data that support the findings of this study are available on request from the corresponding author. The data are not publicly available due to privacy or ethical restrictions.
